# Fair Play a Tavola: a school-based framework integrating nutrition education and anthropometric screening for early identification of excess weight in preschool children

**DOI:** 10.3389/fped.2026.1802064

**Published:** 2026-03-27

**Authors:** Chiara Spiezia, Claudia Di Rosa, Ludovica Di Francesco, Allegra Almanza, Angelica Tomaselli, Giuseppe Stefano Morino, Manuela Trombetta, Yeganeh Manon Khazrai, Laura De Gara

**Affiliations:** 1 Research Unit of Food Science and Human Nutrition, Department of Science and Bio-Technology, Università Campus Bio-Medico di Roma, Roma, Italy; 2 Galatea Comunicazione Fair Play-4U, Roma, Italy

**Keywords:** anthropometric screening, childhood overweight and obesity, dietary habits, early prevention, nutrition education, parental perception, preschool children

## Abstract

**Background:**

Preschool age is a critical yet underexplored period for the early emergence of dietary behaviours and excess weight. Children aged 3–5 years are largely excluded from national nutrition surveillance systems, limiting the timely identification of excess weight and related behavioural patterns. *Fair Play a Tavola* was conceived to integrate nutrition education with anthropometric screening in preschool settings, providing an early epidemiological overview of lifestyle behaviours and excess weight in preschool children.

**Methods:**

A multicenter cross-sectional observational study was conducted between October 2023 and February 2025 in 57 preschools in Lazio, Italy. Approximately 5,000 children aged 3–5 years participated in play-based, multisensory-nutrition-education activities, while anthropometric and questionnaire-based analyses were conducted in a subsample with complete data. Anthropometric measurements and a multilingual parent-reported questionnaire assessed growth parameters, dietary habits, physical activity, screen time, sleep, and family characteristics. BMI categories were defined according to WHO 2006–2007 standards.

**Results:**

The final analytical sample included 1,542 children (48% female; mean age 4.4 ± 0.9 years), of whom 38% showed excess weight (17% at risk of overweight, 14% overweight, 7% obesity). Waist circumference increased progressively across BMI categories (*p* < 0.0001), and higher BMI was associated with higher birth weight and higher parental BMI. Parents frequently underestimated their child's weight status, particularly in overweight categories. Physical activity levels were generally low across the cohort, while children with obesity reported higher video game use. Dietary differences across BMI groups were limited but significant for selected food categories, including fish, eggs, processed meats, and fast food. Across the entire population, adherence to national dietary recommendations (*CREA 2018*) for fruits, vegetables, cereals, and yogurt was consistently low.

**Conclusions:**

Differences in anthropometric measures and selected parent-reported behaviors were observed across BMI-defined categories. In this cross-sectional study, anthropometric screening conducted within preschool settings allowed the descriptive characterization of excess weight and related behavioural patterns at an early developmental stage. Given the partial participation rates and the absence of longitudinal follow-up, findings should be interpreted descriptively. Further longitudinal and intervention-based studies are required to determine long-term impact and preventive effectiveness.

## 1 Introduction

Childhood overweight and obesity are among the main health concerns worldwide. According to the World Health Organization (WHO), in 2024, more than 35 million children under the age of 5 were overweight or obese worldwide, with prevalence increasing in several regions. In the WHO European Region, overweight affects about one in three school-aged children, with countries in southern Europe having the highest rates (1, 2).

In Italy, national surveillance data from the *OKkio alla Salute* system indicate that 19% of children aged 8–9 years are affected by overweight and 9.8% by obesity, corresponding to an overall prevalence of approximately 29% of excess weight (3). These figures place Italy among the countries with the highest prevalence in Western Europe.

Longitudinal studies indicate that excess weight in early childhood substantially increases the likelihood of persistent obesity into adolescence and adulthood and is associated with early cardiometabolic alterations, including adverse body fat distribution, insulin resistance, and elevated blood pressure, reinforcing the need for preventive strategies before school age. However, systematic surveillance data for children under the age of 6 remain limited, resulting in a significant epidemiological gap during the preschool period. The adiposity rebound (4), which typically occurs between the ages of 5 and 6, represents a critical window of development; when it occurs earlier, it is strongly associated with the risk of obesity in later life. Therefore, early identification of anthropometric deviations and associated behavioral patterns before school age is clinically and epidemiologically relevant.

Within this epidemiological context, early childhood represents a pivotal period for the establishment of dietary behaviours, lifestyle patterns, and growth trajectories that may influence long-term health (5). Between 3 and 5 years of age, children undergo rapid physical, cognitive, and emotional development, while progressively forming food preferences, eating routines, and self-regulatory behaviours related to food intake (6). This developmental stage is frequently characterized by food neophobia and selective eating, which may reduce dietary variety and compromise the intake of essential nutrients. When persistent, these behaviours can favour a preference for energy-dense, nutrient-poor foods and a reduced consumption of fruits, vegetables, and fiber-rich options, contributing to early nutritional imbalances and increased metabolic risk (7–9). At this stage, these factors do not translate into measurable clinical outcomes but rather define early behavioural and nutritional contexts in which excess weight may emerge.

Such imbalances may influence the timing of adiposity rebound, the physiological point at which body mass index begins to increase after an early childhood nadir. An early adiposity rebound (EAR), generally occurring before the age of 5 years, and a very early adiposity rebound (VEAR), occurring before 3 years of age, have been consistently identified as predictors of later overweight, obesity, and cardiometabolic alterations (10, 11). Understanding behaviours that precede or accompany this sensitive phase may help contextualize early risk patterns, even when longitudinal growth trajectories are not directly assessed. At the same time, several studies have documented a progressive departure from the traditional Mediterranean dietary pattern toward more Westernized eating habits, characterized by lower consumption of fresh and minimally processed foods and increased intake of sugar-and fat-rich products. This nutritional transition is already evident in early childhood and has been associated with rising rates of overweight and obesity. Conversely, regular consumption of vegetables and the use of extra-virgin olive oil, key components of the Mediterranean diet, have been identified as protective factors against excessive weight gain (12–14).

To address these early risks, educational strategies implemented during the preschool years may promote food exploration through enjoyable and multisensory experiences. Sensory-based activities involving touch, smell, taste, and simple food preparation can enhance familiarity with foods, stimulate curiosity, and reduce food neophobia and selective eating. In contrast, coercive feeding practices or excessively permissive approaches have been shown to be ineffective and may contribute to the development of food aversions and maladaptive eating behaviours over time (11, 12).

However, dietary education alone may be insufficient to fully address early metabolic risk, as eating patterns develop within a broader lifestyle context that includes physical activity, sedentary behaviours, and sleep patterns (15, 16). Within this context, insufficient levels of physical activity and increasing exposure to sedentary patterns, including screen-based entertainment, have been reported even among preschool-aged children and are associated with unfavourable energy balance and weight gain. Sleep duration and regularity also contribute to metabolic regulation and appetite control, with shorter or irregular sleep patterns being linked to a higher obesity risk (16).

These behaviours are strongly influenced by the family environment, which represents the primary context in which eating habits and lifestyle patterns are modelled, learned, and reinforced. Parental weight status, educational level, and perceptions of their child's body weight have been shown to affect children's behaviours and the early recognition of excess weight (17). At this age, differences in dietary and lifestyle behaviours are often subtle, yet potentially meaningful when observed at a population level.

Despite the recognized importance of early prevention, preschool-aged children are not systematically included in national nutrition surveillance systems. In Italy, the *OKkio alla Salute* surveillance system (3), provides extensive data on overweight, obesity, dietary habits, and physical activity among primary school children aged 6–10 years, but does not cover younger children aged 3–5 years. Consequently, national surveillance data on nutritional status and lifestyle behaviours during this early developmental stage remain limited, despite its high plasticity and responsiveness to educational interventions. Targeted preschool-based initiatives may therefore complement existing surveillance frameworks by extending observation to earlier stages of life, when risk behaviours begin to emerge.

Unlike many preschool studies focusing on either nutrition education or anthropometric screening alone, Fair Play a Tavola was conceived as an integrated, school-based framework combining structured nutrition education, systematic anthropometric assessment, and family feedback.

The primary aim of this study was to implement a structured nutrition education program within preschool settings, engaging more than 5,000 children aged 3–5 years through a playful and multisensory approach designed to foster a positive relationship with food.

Secondary aims included describing dietary habits and lifestyle behaviours of children and their families, collecting anthropometric measurements as part of an early screening strategy, and exploring differences in nutritional and behavioural patterns according to weight status. An additional objective was to actively involve families through feedback and counselling activities, with the aim of increasing awareness of early nutritional and behavioural challenges during the preschool years and their potential long-term implications.

Overall, the study aimed to contextualize early dietary and lifestyle behaviours within a developmental phase characterized by high plasticity, reinforcing preschool age as a critical window for excess weight prevention.

## 2 Materials and methods

### 2.1 Study design and participants

The Fair Play a Tavola project was conducted between October 2023 and February 2025 as a multicenter, cross-sectional observational study with a descriptive aim, conducted in preschool settings and including a contextual nutrition education component.

The study was implemented in collaboration with public and private preschools located in Rome and surrounding municipalities within the Lazio region, following a standardized protocol to ensure methodological consistency across participating centres.

**Figure 1:**
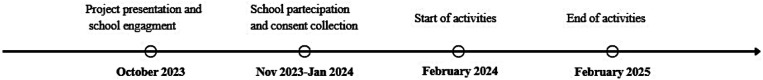
Timeline of the Fair Play a Tavola project, from initial school engagement to completion of educational and screening activities (October 2023–February 2025).

Preschools were recruited through direct institutional contact by the research team. In October 2023, the project was formally presented to school principals and teaching staff, outlining its objectives, methodology, operational procedures, and ethical framework. Recruitment was facilitated through established professional networks across several municipal districts, allowing dissemination of project information to eligible preschools. Participation was voluntary, and no probability-based sampling strategy was applied (Figure 1).

All schools contacted agreed to participate (57/57). No comprehensive register of all eligible preschools in the Lazio region was used as a sampling frame; therefore, it is not possible to calculate a formal response rate at the school level relative to the total number of eligible schools. No predefined inclusion or exclusion criteria were established at the school level, except for the admission of children in the target preschool age group (3–5 years). Given the voluntary nature of recruitment, the participating schools constitute a convenience sample.

Following school agreement, principals distributed written information letters and informed consent forms to families, together with details regarding the organization of educational activities and anthropometric assessments. Children were eligible for participation upon provision of written informed consent by a parent or legal guardian.

Each participant was assigned a unique alphanumeric identification code linked to a QR-based system, allowing a pseudonymized linkage between questionnaire data and anthropometric measurements. No directly identifiable personal data were collected at any stage, and all procedures were conducted in accordance with the EU General Data Protection Regulation (GDPR 2016/679).

Data collection included parent-reported questionnaires, anthropometric screening (weight, height, and waist circumference), and the implementation of age-appropriate educational activities. Feedback sessions and individual consultations were subsequently offered to parents, as described in the corresponding sections below. All activities were carried out by trained nutritionists following standardized procedures to ensure uniform implementation across schools.

### 2.2 Questionnaire

The parent-reported questionnaire was developed for research purposes by adapting selected items from two existing instruments: the questionnaire used in the national surveillance system *OKkio alla Salute* (Istituto Superiore di Sanità) and the validated KIDMED index for adherence to the Mediterranean diet (3, 18). Items were contextualized for the preschool age range (3–5 years) and designed to capture habitual dietary and lifestyle behaviours relevant to early childhood.

The original version was developed in Italian and subsequently translated into English, French, Spanish, and Arabic to ensure accessibility among families of diverse linguistic backgrounds. Translations were carried out by bilingual collaborators and native speakers familiar with the cultural context of the target populations. The translated versions were reviewed by the research team to ensure conceptual coherence and semantic clarity, and minor linguistic refinements were introduced where necessary; no substantial cultural adaptations of item content were required.

Formal forward–backward translation procedures and cross-cultural psychometric validation were not performed, in line with the descriptive and exploratory nature of the study. Prior to large-scale administration, the questionnaire was pilot-tested in a small subsample of families to assess clarity and comprehensibility.

Although not formally validated, the content and structure of the questionnaire were reviewed by a paediatrician and a nutritionist with expertise in paediatric nutrition to ensure face and content validity.

The final questionnaire consisted of 52 items grouped into six thematic domains (Table 1). It was administered online via Google Forms, optimized for smartphone and tablet use. Automatic logic checks were implemented to prevent inconsistent responses, and duplicate entries were resolved by retaining the most complete record.

**Table 1:** Structure of the parent-reported questionnaire. The questionnaire included 52 items grouped into six sections: structured physical activity, use of technology devices, sleep habits, eating habits, family assessment, and neonatal information.

Questionnaire section	Questionnaire items
Section I: structured physical activity q.1	Weekly frequency of structured physical activity (days/week)
Section II: use of technology devices q. 2–6	Average time spent using video games or watching TV (school days and weekends) Presence of a TV in the child's bedroom
Section III: sleep habits q.7–8	Bedtime Wake-up time
Section IV: eating habits q. 9–35	Breakfast consumption. Daily water intake. Weekly frequency of consumption of major food groups (fruits, vegetables, legumes, fish, meat, dairy products, cereals and bakery products, fats and condiments, snacks, beverages, fast food)
Section V: family assessment q. 36–39 q. 44–52	Parent's perception of the child's weight status. Educational attainment. Employment type (part-time/full-time). Weight and height of biological parents (BMI calculation). Family medical conditions. Child's medical conditions Presence of allergies/intolerances.
Section VI: neonatal information q.40–43	Type of delivery: natural/caesarean. Birth details: full-term/preterm/post-term. Birth weight Type of feeding: breastfeeding/formula feeding.

### 2.3 Nutrition education activities

The educational component of the Fair Play a Tavola project was specifically designed for preschool children and was implemented as a standardized school-based activity integrated within the broader screening initiative. The program was based on a play-based and multisensory learning approach consistent with early childhood education principles (8, 19).

Educational sessions were delivered by trained nutritionists during regular school hours according to a predefined structure to ensure methodological consistency across participating schools. Each class received one structured session of approximately 60 min.

Activities were tailored to age group (3, 4, and 5 years) and included interactive games, guided motor-response exercises, food recognition and categorization tasks, and multisensory workshops (“look, touch, smell”). Age-specific educational activities and corresponding learning objectives are summarized in Table 2.

**Table 2:** Age-specific educational activities and learning objectives of the nutrition education program.

Age group	Activity	Education objective
3 years	Recognition of fruits and vegetables with motor response (stand up for vegetables, stay seated for fruits).	Promote basic food categorization skills and support sensorimotor engagement through simple action–response associations.
4 years	Identification of displayed food items with corresponding motor response.	Expand food-related vocabulary and reinforce correct association between foods and their respective categories.
5 years	“Find the odd one out” (identification of the food item that does not belong to the presented category).	Develop higher-order classification skills, observation abilities, and cognitive comparison between food items.
All age groups	Sensory workshop “Look, touch, smell”.	Enhance familiarity with fruits and vegetables through multisensory exploration and reduce food neophobia.
	Sticker classification (place the image in the fruit or vegetable column).	Consolidate food recognition and categorization skills while promoting active participation and cooperative learning.

Group size was adapted to classroom composition and daily attendance, generally involving approximately 10–20 children per session to maintain age-appropriate engagement and interaction. When necessary, smaller classes were combined, and multiple nutritionists allowed parallel delivery across classrooms within the same school.

In addition to classroom activities, participating schools received a standardized educational kit including animated videos developed in collaboration with the Eli Editorial Group and an illustrated booklet translated into Italian Sign Language (LIS), produced with the support of Autostrada per l'Italia and CREI—Cooperativa Sociale Interpreti LIS. These materials were intended to support continuity between school and home environments.

### 2.4 Anthropometric screening

Anthropometric screening was conducted with parental consent to measure weight, height, and waist circumference using standardized equipment (SECA scales, stadiometers, and non-elastic measuring tapes). Children were measured wearing light indoor clothing and without shoes to ensure accuracy. Each measurement was taken twice by trained independent operators, and the average value was recorded to minimize inter-observer variability. Body mass index (BMI) was calculated as weight (kg)/height^2^ (m^2^) and interpreted according to World Health Organization reference growth standards: WHO 2006 curves for children aged 2–5 years (0–60 months) and WHO 2007 curves for children aged ≥5 years (61–228 months) (20, 21).

### 2.5 Parent feedback sessions

Feedback sessions with parents were conducted in the afternoon by a multidisciplinary team consisting of nutritionists and a paediatrician. The sessions were structured to ensure methodological consistency across schools and to facilitate standardized communication of results to families.

Each session included:

(i) a summary presentation of educational activities and aggregate results of questionnaires and anthropometric assessments,
(ii) a detailed explanation of data collection procedures and interpretation of BMI and percentile-based growth indicators,
(iii) individual consultations, during which parents received personalized, evidence-based recommendations aimed at optimizing children's eating habits and addressing specific nutritional or behavioural concerns.

These sessions were intended to promote parental awareness and involvement in nutrition-related health education, fostering continuity between the school and home environments and supporting the translational component of the project.

### 2.6 Ethical approval

The study was conducted in accordance with the principles of the Declaration of Helsinki and was approved by the Ethics Committee of the Campus Bio-Medico University of Rome (approval code 131/21). Written informed consent was obtained from all parents or legal guardians prior to participation.

### 2.7 Statistical analysis

Statistical analyses were performed using GraphPad Prism software, version 10.4.1. Normality of continuous variables was assessed using the Shapiro–Wilk test. As most variables were not normally distributed (*p* < 0.0001), data were summarized using descriptive statistics appropriate to variable type.

Questionnaire items were analyzed according to their measurement scale. Ordinal variables, including the weekly frequency of structured physical activity and food consumption frequencies, were summarized using median values with interquartile range (IQR). Screen-based behaviours were described using mean ± standard deviation (SD) of ordinal scores for descriptive purposes only, reflecting overall exposure, while between-group comparisons were performed using non-parametric methods. For questions 44–47, parental BMI was calculated as weight (kg)/height^2^ (m^2^). For all other items, categorical responses were assigned ordinal scores, and median values were determined for each weight-status group (A–D). Between-group comparisons for continuous or ordinal variables were conducted using the Kruskal–Wallis test followed by Dunn's *post-hoc* test. Effect size for Kruskal–Wallis analyses was estimated using epsilon-squared (*ε*^2^). Categorical variables, including dichotomous items, were analyzed using the Chi-square (*χ*^2^) test. To evaluate adherence to national dietary recommendations, weekly consumption frequencies were compared with the CREA Guidelines for Healthy Eating (2018). Reference values for children aged 2–3 and 4–6 years were averaged to obtain a single benchmark applicable to the 3–5-year age range. Food categories were considered adherent when reported consumption met or exceeded the corresponding reference.

Given the descriptive and exploratory nature of the study, analyses were conducted on complete cases only, without imputation of missing data, to avoid introducing additional assumptions. No multilevel modeling was applied; therefore, results should be interpreted as descriptive associations rather than causal relationships.

All tests were two-sided, and *p*-values <0.05 were considered statistically significant.

## 3 Results

### 3.1 Study implementation and participant flow

The Fair Play a Tavola project involved a total of 5,076 preschool children from 57 participating schools across the Lazio region. Although schools were located in areas with potentially different social stratification, no formal area-level socioeconomic indicators were collected.

All 57 participating schools completed the planned educational and screening activities according to the predefined protocol. One structured nutrition education session of approximately 60 min was delivered to each participating class during regular school hours by trained nutritionists. Group size was adapted to classroom composition and daily attendance, generally involving 10–20 children per session to ensure age-appropriate engagement. No schools withdrew after initial agreement.

Following completion of the screening phase, one structured parent feedback meeting was organized in each participating school (57/57). These meetings were conducted by a multidisciplinary team including nutritionists and a pediatrician and included presentation of aggregated questionnaire and anthropometric results, explanation of BMI classification and growth indicators, and general informational messages related to healthy growth and lifestyle behaviours. Parents were given the opportunity to ask questions or seek clarification during or after the session. Quantitative indicators of parental attendance or number of individual consultations were not systematically recorded.

Anthropometric screening and questionnaire-based analyses were conducted only among children with parental authorization and complete data.

Parental authorization for anthropometric screening was obtained for 2,444 children (48%), while 2,139 parents (42%) completed the questionnaire; among these, 1,636 children had both anthropometric measurements and completed questionnaires.

Of this subgroup, 1,545 children (94%) were within the predefined target age range (3–5 years and 11 months; 36–71 months), including 742 girls (48%), whereas 91 children (6%) were outside the age range (27 aged 2 years and 64 aged ≥6 years).

**Figure 2:**
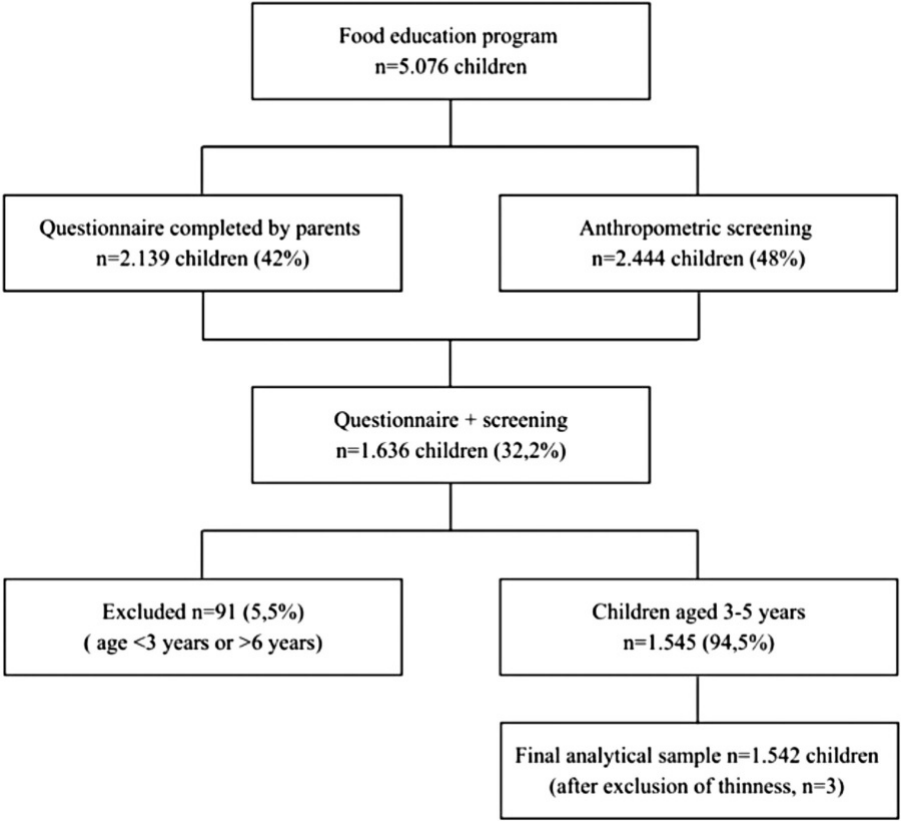
Flow chart of participant selection.

After exclusion of three children classified as underweight (0.2%), the final analytical sample consisted of 1,542 children (739 girls, 48%) with a mean age of 4.47 ± 0.91 years (Figure 2).

### 3.2 Anthropometric findings

#### 3.2.1 Study population characteristics

The mean anthropometric characteristics of the final analytical sample are reported in Table 3.

**Table 3:** Anthropometric characteristics of study population.

Weight (kg)	Height (cm)	BMI (kg/m^2^)	BMI *z*-score	Waist circumference (cm)
18.59 ± 3.57	105.66 ± 7.79	16.52 ± 1.68	0.77 ± 1.02	52.97 ± 4.50

**Figure 3:**
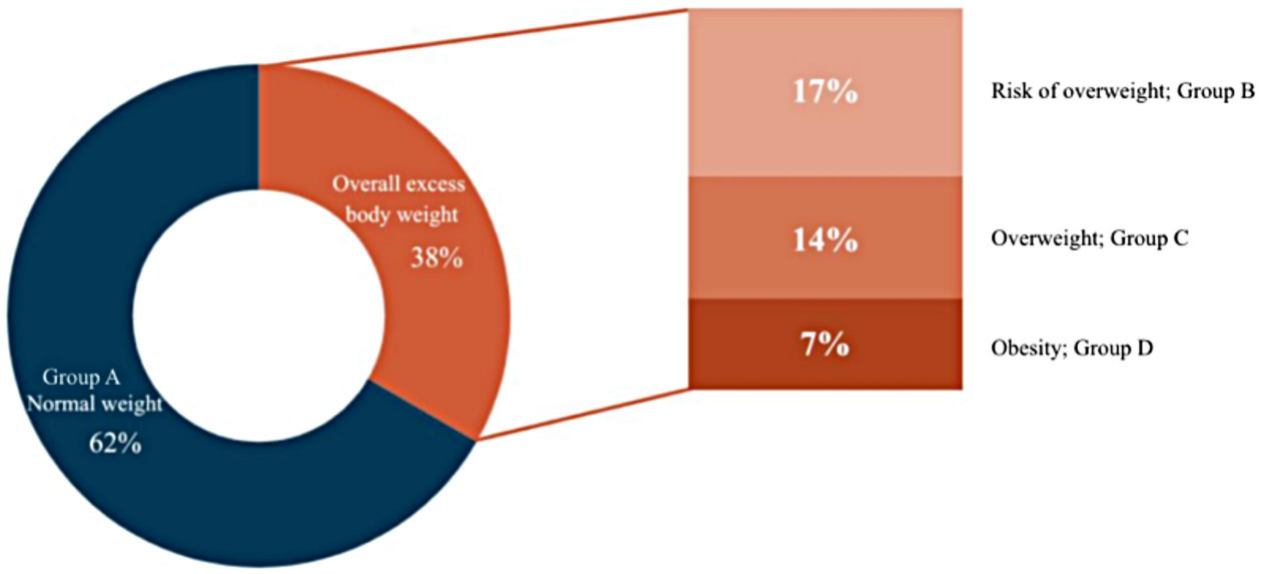
Distribution of weight status in the study population.

According to the WHO 2006 and 2007 reference standards, children were classified into four weight-status groups (Figure 3): Group A (normal weight; *n* = 964, 62%), Group B (at risk of overweight; *n* = 267, 17%), Group C (overweight; *n* = 210, 14%), and Group D (obesity; *n* = 101, 7%).

#### 3.2.2 Waist circumference

**Figure 4:**
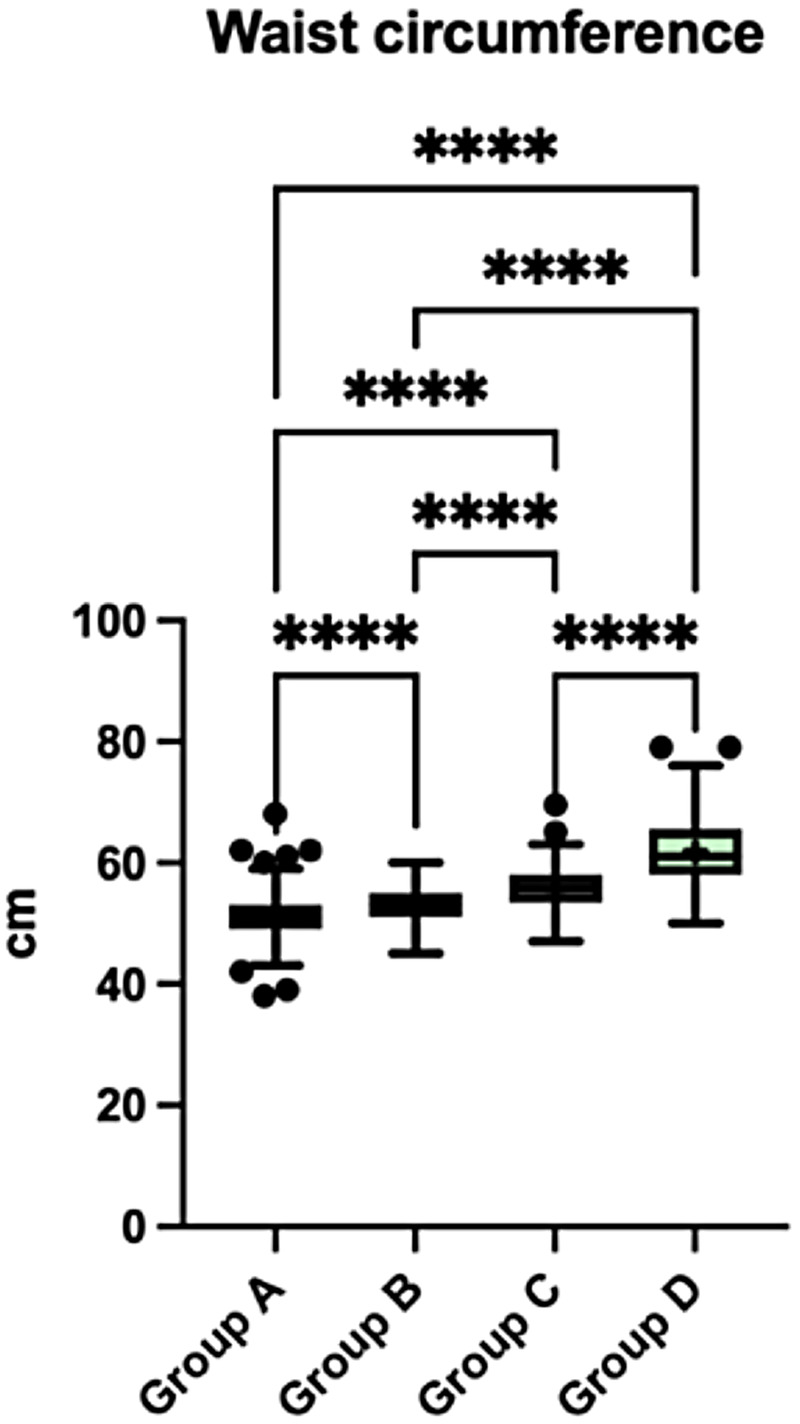
Waist circumference across the four weight-status groups (A–D). Boxplots display the median and interquartile range, with whiskers indicating the minimum and maximum values. Crosses (+) represent mean values, and dots indicate outliers. Statistically significant between-group differences are indicated in the figure (*p* < 0.05). Data were analysed using the Kruskal–Wallis test followed by Dunn's *post-hoc* test with correction for multiple comparisons.

Waist circumference increased progressively across weight-status groups, from 51.0 ± 3.0 cm in Group A to 61.8 ± 5.8 cm in Group D (*p* < 0.0001). All pairwise comparisons between groups were statistically significant (Figure 4).

### 3.3 Questionnaire results

To facilitate interpretation and improve clarity, questionnaire results are presented by thematic domain rather than by individual question number. Overall, differences across weight-status groups were limited and mainly concerned selected lifestyle behaviours and specific food categories.

#### 3.3.1 Structured physical activity

**Figure 5:**
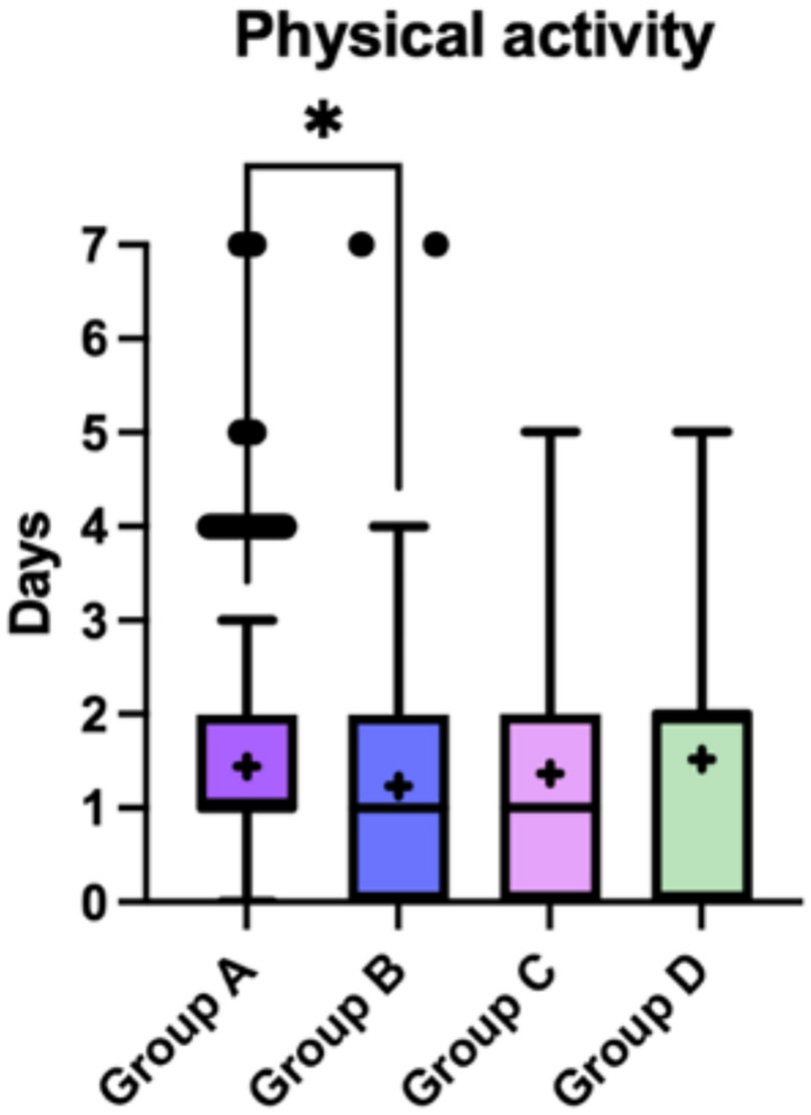
Weekly frequency of structured physical activity across the four weight-status groups (A–D). Boxplots display the median and interquartile range, with whiskers indicating the minimum and maximum values. Crosses (+) represent mean values, and dots indicate outliers. A statistically significant difference was observed between Groups A and B (*p* < 0.05). Data were analysed using the Kruskal–Wallis test followed by Dunn's *post-hoc* test with correction for multiple comparisons. Scores represent the number of days per week of structured physical activity (range: 0–7 days).

Structured physical activity was generally low across the study population, with most children engaging in organized activity fewer than 2 days per week. Distributions overlapped across weight-status groups, with a statistically significant difference observed only between Groups A and B (*p* = 0.021) (Figure 5).

#### 3.3.2 Use of electronic devices

##### 3.3.2.1 Video game use

**Figure 6:**
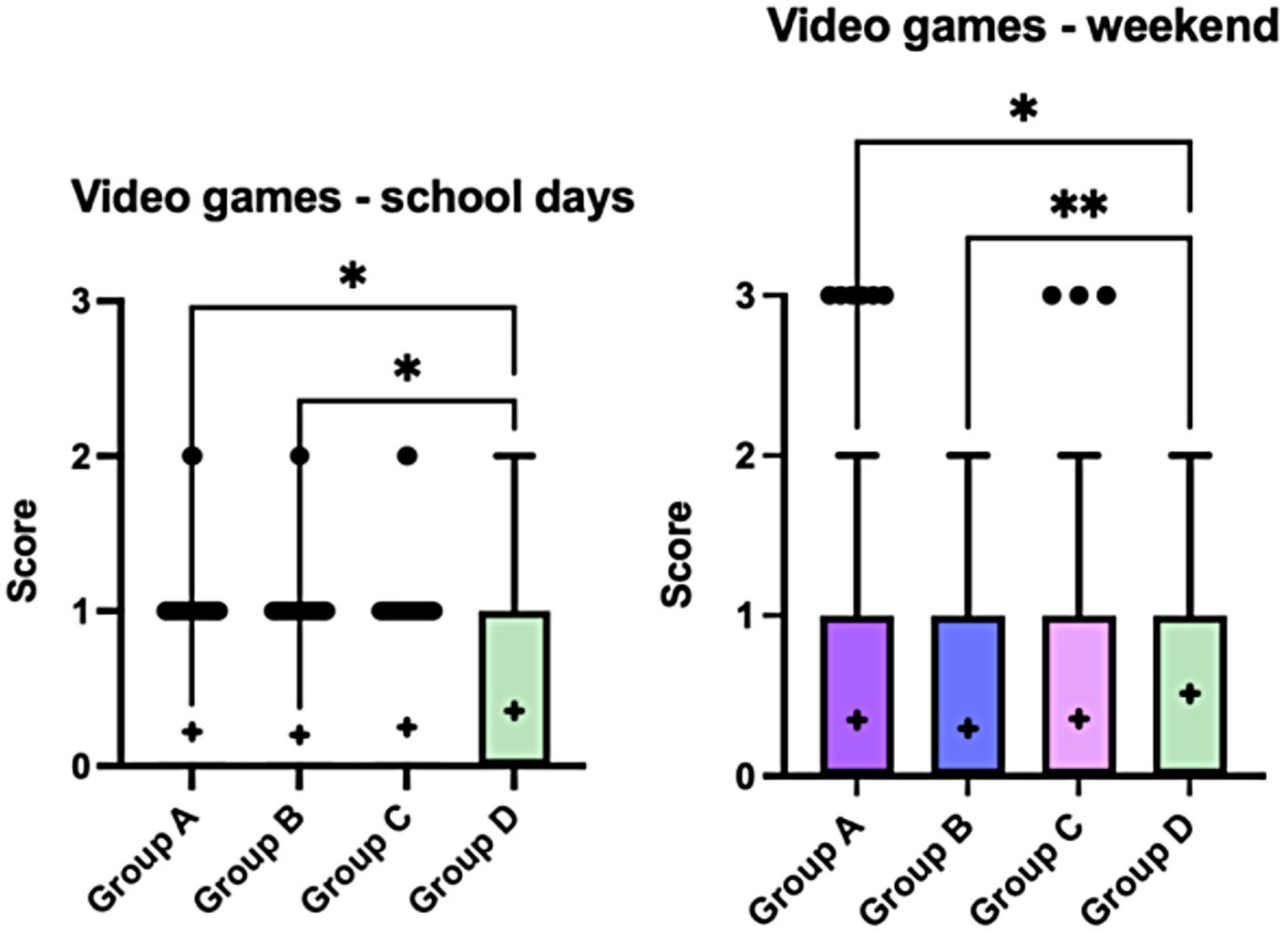
Daily frequency of video game use on school days and weekends across the four weight-status groups (A–D). Boxplots display the median and interquartile range, with whiskers indicating the minimum and maximum values. Crosses (+) represent mean values, and dots indicate outliers. Statistically significant between-group differences are indicated in the figure (*p* < 0.05). Data were analysed using the Kruskal–Wallis test followed by Dunn's *post-hoc* test with correction for multiple comparisons. Scores represent ordinal categories of average daily video game use (0 = no use; 1 = 1–3 h/day; 2 = 3–5 h/day; 3 ≥ 5 h/day).

During school days, video game use was generally low across all weight-status groups, with most children reporting no use. Nevertheless, children in Group D showed significantly higher video game use compared with Groups A and B (*p* = 0.044 and *p* = 0.032, respectively), reflected by higher mean score values and a lower proportion of non-users (67% vs. 79% in Group A and 81% in Group B). During weekends, video game use increased slightly across all groups while maintaining a similar distribution. Group D again reported higher use compared with Groups A and B (*p* = 0.039 and *p* = 0.009, respectively), mainly driven by a lower proportion of non-users (57% in Group D vs. 70% in Group A and 74% in Group B) (Figure 6).

##### 3.3.2.2 Television viewing

Television viewing habits did not differ significantly among weight-status groups. Watching television for 1–3 h per day was the most reported behaviour, observed in 75.8% of children on school days and 84% during weekends. Approximately one quarter of the sample reported having a television in the child's bedroom.

#### 3.3.3 Sleep habits

Sleep patterns were broadly similar across all weight-status groups. Approximately half of the children went to bed between 9:00 and 10:00 p.m., and about 69% woke up between 7:00 and 8:00 a.m. No statistically significant differences were observed among groups.

#### 3.3.4 Dietary habits

No statistically significant differences were observed across weight-status groups for most dietary habits.

Breakfast was consumed daily by 77% of children.

Reported daily water intake was approximately 1.5 L/day in Groups B, C, and D, whereas children in Group A reported a lower intake, although this difference was not statistically significant.

Regarding food frequency:

- Cereals and bakery products: Pasta or other cereals were consumed 4–6 times per week by 28% of children, and bread by 27.8%. Pizza consumption was reported less than once per week by 51.5% of participants.
- Fruits and vegetables: Vegetable intake was mainly reported as 1–3 times per week in Groups A and D and 4–6 times per week in Groups B and C. Fresh fruit was consumed 4–6 times per week by 30.6% of children. Nut consumption was generally low, with less than one serving per week reported in Groups A and B and no consumption reported in Groups C and D.
- Milk and dairy products: Half of the children consumed cow's milk daily. Yogurt, fresh cheese, and aged cheese were most consumed 1–3 times per week, reported by 33.3%, 58.2%, and 44% of children, respectively.
- Protein sources: White meat, red meat, and legumes were generally consumed 1–3 times per week by 64.4%, 68.7%, and 67% of children, respectively.
- Fats and condiments: Extra-virgin olive oil was the predominant source of fat, used by 99% of children. Among these, 64% reported daily consumption, with 27% reporting use once per day. Seed oil and butter were infrequently consumed, with 67% and 52.7% of children, respectively, reporting no habitual use.
- Beverages and snacks: Fruit juices were consumed 1–3 times per week by 36% of children, while 65% reported never consuming sugary beverages. Salty and sweet snacks were consumed 1–3 times per week by 44% of children, and chocolate by 42%.

**Figure 7:**
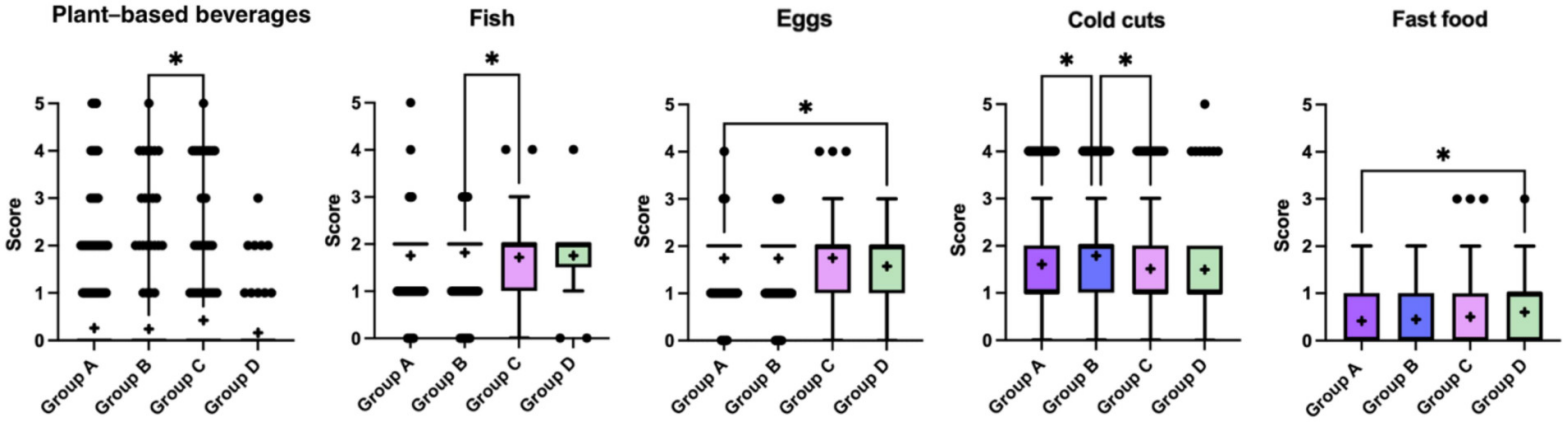
Weekly consumption frequency of selected food categories (plant-based beverages, fish, eggs, cold cuts, and fast food) across the four weight-status groups (A–D). Boxplots display the median and interquartile range, with whiskers indicating the minimum and maximum values. Crosses (+) represent mean values, and dots indicate outliers. Statistically significant between-group differences are indicated in the figure (*p* < 0.05). Data were analysed using the Kruskal–Wallis test followed by Dunn's *post-hoc* test with correction for multiple comparisons. Consumption frequency was coded as an ordinal score: 0 = never; 1 = less than once per week; 2 = 1–3 days per week; 3 = 4–6 days per week; 4 = once daily; 5 = more than once daily.

Statistically significant differences among weight-status groups were observed only for a limited number of food categories, namely plant-based beverages, fish, eggs, cold cuts, and fast food (Figure 7).

- Plant-based beverages: Consumption was limited across all weight-status groups, with more than 80% of children reporting no intake. A statistically significant difference was observed between Groups B and C (*p* = 0.028), with a higher proportion of non-consumers in Group B (91%) compared with Group C (82.4%).
- Fish: Fish consumption was predominantly reported at frequencies up to 1–3 times per week across all weight-status groups. A statistically significant difference was observed between Groups B and C (*p* = 0.023), with a higher proportion of children reporting fish intake less than once per week in Group C (25.3% vs. 11.6%) compared with Group B.
- Eggs: Egg consumption was mainly reported at frequencies of 1–3 times per week across all weight-status groups. A statistically significant difference was observed between Groups A and D (*p* = 0.027), with Group D showing a higher proportion of children reporting consumption less than once per week (30.7% vs. 19.7%).
- Cold cuts: Children in Group B reported higher consumption, most commonly 1–3 times per week, compared with more occasional consumption (<1 time per week) in Groups A and C (Group B vs. Group A: *p* = 0.048; Group B vs. Group C: *p* = 0.046).
- Fast food: Most children reported never consuming fast food. A higher proportion of non-consumers was observed in Group A compared with Group D (61.6% vs. 48.5%; *p* = 0.025).

#### 3.3.5 Adherence to national dietary guidelines (CREA guidelines for healthy eating 2018)

Adherence to national dietary recommendations was evaluated in the overall study population by comparing reported consumption frequencies with the CREA Guidelines for Healthy Eating (2018) (22), independently of weight-status categories, to provide a population-level assessment of dietary patterns.

As shown in Table 4, adherence to recommended intake frequencies was generally low across several core food groups, including fruits, vegetables, cereals, and yogurt. For all dietary categories, statistically significant differences between reported intakes and guideline recommendations were observed (*p* < 0.05), except for fast food consumption, indicating a widespread discrepancy between observed eating habits and national targets.

**Table 4:** Comparison between reported dietary intake and CREA guidelines for healthy eating (2018) in the overall study population.

Food group	Recommended frequencies were derived by averaging CREA guidelines for children aged 2–3 and 4–6 years to match the 3–5-year age range of the study population.	Observed adherence (% of children meeting recommended frequency)	*P*-values refer to comparisons between observed intake frequencies and CREA recommended frequencies using contingency tables.
Carbohydrates	Pasta/cereals: twice daily	Pasta/cereals: 14%	<0.0001
	Bread: twice daily	Bread: 7%	
	Pizza: once per week	Pizza: 57%	
Vegetables and fruits	Vegetables: twice daily	Vegetables: 13%	<0.0001
	Fresh fruit: 2–3 times daily	Fresh fruit: 21%	
	Nuts: 3 times per week	Nuts: 16%	
Milk and dairy products	Milk: daily	Milk: 50%	<0.0001
	Yogurt: 4–5 times per week	Yogurt: 9%	
	Fresh cheese: 2–3 times per week	Fresh cheese: 58%	
	Aged cheese: 2–3 times per week	Aged cheese: 44%	
Protein sources	Fish: 3 times per week	Fish: 75%	<0.0001
	White meat: 2 times per week	White meat: 64%	
	Red meat: once per week	Red meat: 24%	
	Eggs: 2 times per week	Eggs: 73%	
	Legumes: 3 times per week	Legumes: 67%	
	Processed meats: occasional	Processed meats: 54%	
Fats and condiments	Extra-virgin olive oil: daily	EVO oil: 64%	<0.0001 (extra virgin oil)
	Seed oil/butter: occasional	Seed oils: 93%	*p* = 0.0140 (seed oil)
		Butter: 88%	*p* = 0.0003 (butter)
Discretionary foods	Sugary drinks, fast food, snacks, chocolate: occasional	Sugary drinks: 88%	*p* = 0.0003 (sugary drinks)
		Fruit juices: 46%	*p* ≤ 0.0001 (fruit juices, snacks, chocolate)
		Snacks: 16%	ns (fast food; observed frequency aligned with recommendations)
		Chocolate: 34%	
		Fast food: 96%	

Cheese intake recommendations were defined according to CREA age-specific guidelines. For children aged 2–3 years, recommended portions ranged from 20 to 30 g per serving depending on fat content; for children aged 4–6 years, recommended portions ranged from 20 to 40 g. Nut portion sizes are not defined for children aged 2–3 years; therefore, recommendations refer only to those aged 4–6 years. These age-specific values were used to define adherence in the study population.

Adherence was lower for carbohydrates and plant-based foods, with a limited proportion of children meeting the recommendations for pasta and cereals, bread, vegetables, fresh fruit, and nuts. For dairy products, adherence differed by item: moderate compliance was observed for fresh and aged cheeses, whereas yogurt consumption showed lower adherence. In contrast, adherence to recommended frequencies for several protein sources, including fish, eggs, legumes, and white meat, was higher, while intake of red meat exceeded the recommended frequency in a large proportion of the sample.

Regarding fats and condiments, more than half of the study population reported daily use of extra-virgin olive oil, whereas seed oils and butter were predominantly consumed occasionally, consistent with guideline indications. For discretionary foods, most children reported occasional consumption of sugary drinks and fast food, while reported intake of fruit juices, snacks, and chocolate exceeded recommended frequencies in a substantial proportion of participants.

#### 3.3.6 Family assessment

**Figure 8:**
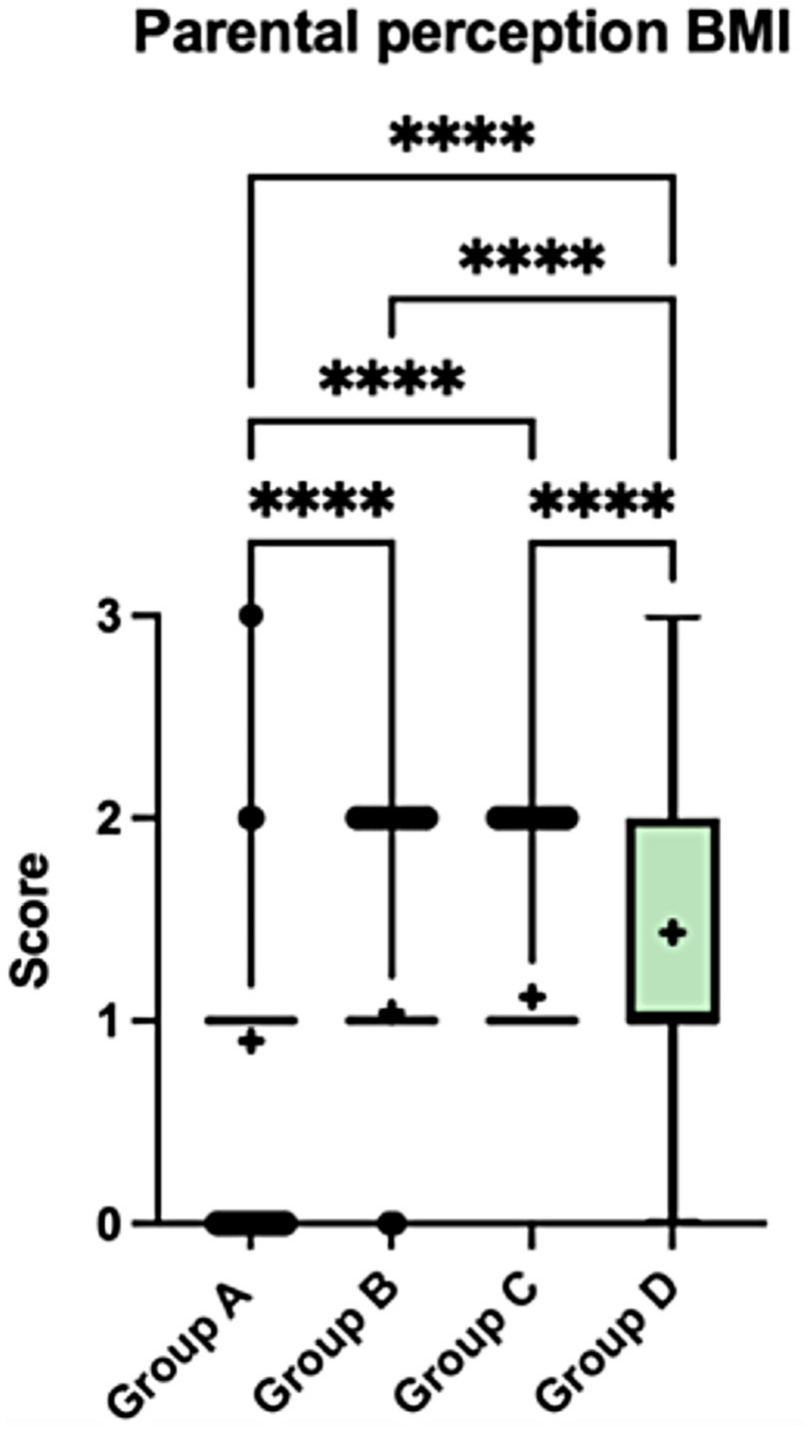
Parental perception of child weight status across the four-child weight-status groups (A–D). Boxplots display the median and interquartile range, with whiskers indicating the minimum and maximum values. Crosses (+) represent mean values, and dots indicate outliers. Statistically significant between-group differences are indicated in the figure (*p* < 0.05). Data were analysed using the Kruskal–Wallis test followed by Dunn's *post-hoc* test with correction for multiple comparisons. Parental perception was coded as an ordinal score: 0 = underweight; 1 = normal weight; 2 = slightly overweight; 3 = markedly overweight.

Parents frequently underestimated their child's weight status, with most parents classifying their child as normal weight across all BMI categories. In Group D, although some parents reported higher perception scores, these were mainly indicative of a “slightly overweight” classification rather than obesity, suggesting limited awareness even in the presence of confirmed obesity. Significant discrepancies between perceived and actual weight status were observed between Groups A and B, A and C, A and D, B and D, and C and D (all *p* < 0.0001), while no significant difference was observed between Groups B and C (Figure 8).

**Figure 9:**
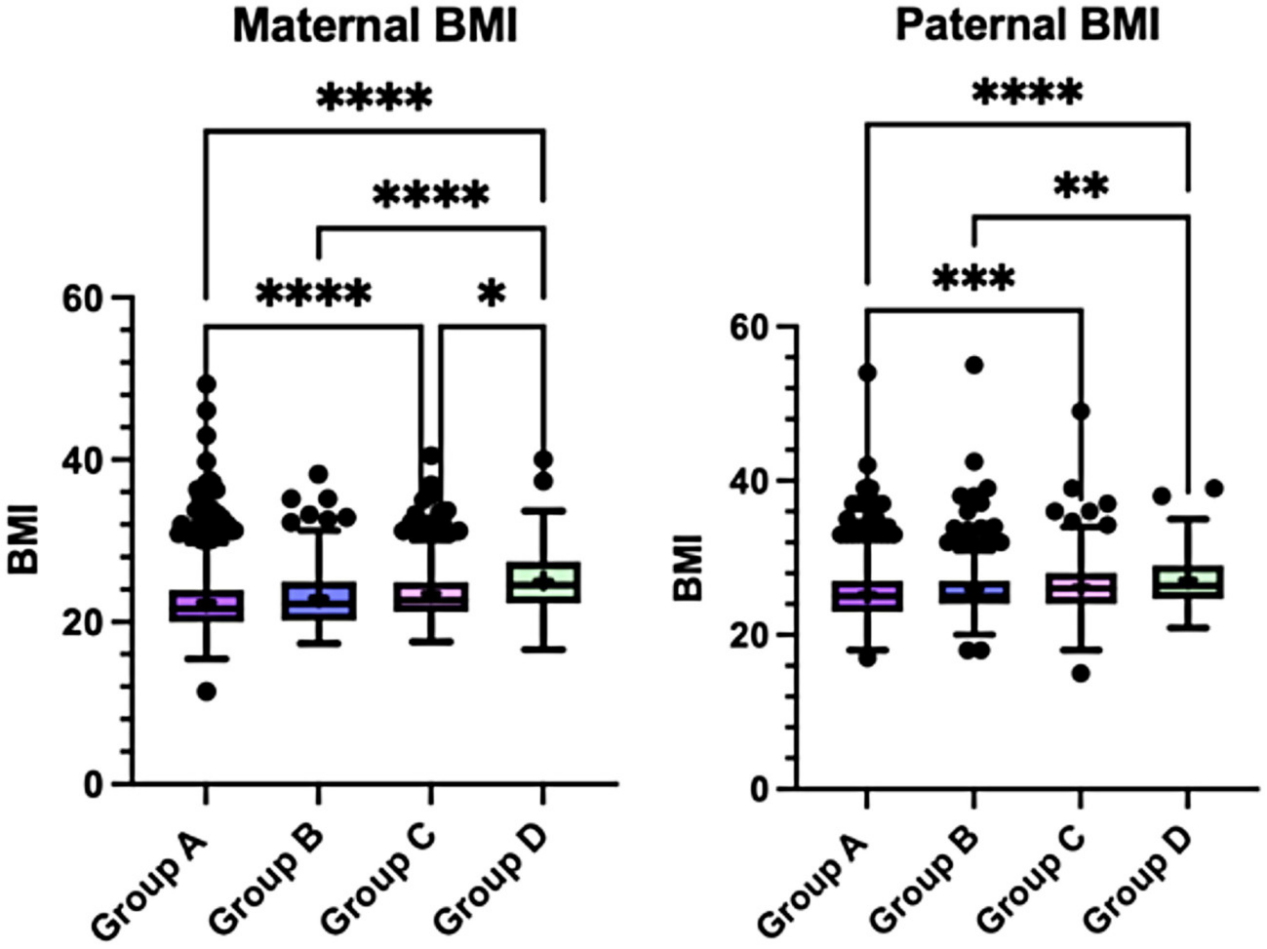
Maternal and paternal BMI across the four child weight-status groups (A–D). Boxplots display the median and interquartile range, with whiskers indicating the minimum and maximum values. Crosses (+) represent mean values, and dots indicate outliers. Statistically significant between-group differences are indicated in the figure (*p* < 0.05). Data were analysed using the Kruskal–Wallis test followed by Dunn's *post-hoc* test with correction for multiple comparisons.

Maternal and paternal BMI distributions showed a progressive shift toward higher values across child weight-status groups, with the highest values observed among parents of children with obesity (all *p* < 0.0001) (Figure 9).

No statistically significant differences were observed across weight-status groups with respect to parental education, employment status, or family medical history.

Nevertheless, a descriptive gradient in parental educational attainment was observed across groups. In Groups A and C, families most frequently reported a heterogeneous educational profile, with one parent holding a university degree and the other a secondary school diploma. In Group B, both parents more commonly held a university degree, whereas in Group D a higher proportion of families reported both parents holding a secondary school diploma.

Employment status was comparable across groups, with approximately half of parents reporting full-time employment outside the home.

Most children (92%) were reported to have no allergies or intolerances. Among the remaining 8%, approximately half of the reported conditions were food related. The most frequently reported food-related conditions were lactose intolerance (29%), fruit and vegetable allergies (18%), and celiac disease (17%), followed by allergies to nuts (11%), eggs (7%), milk protein (5%), legumes (5%), and favism (5%). Histamine intolerance (2%) and fish or salmon allergy (1%) were rarely reported. No statistically significant differences in the prevalence of allergies or intolerances were observed across weight-status groups.

#### 3.3.7 Neonatal and perinatal information

Neonatal and perinatal characteristics were largely homogeneous across groups. Overall, 59% of children were born by vaginal delivery and 82.4% were born at term. Breastfeeding duration was generally longer than 6 months, although a shorter duration was observed in Group D.

**Figure 10:**
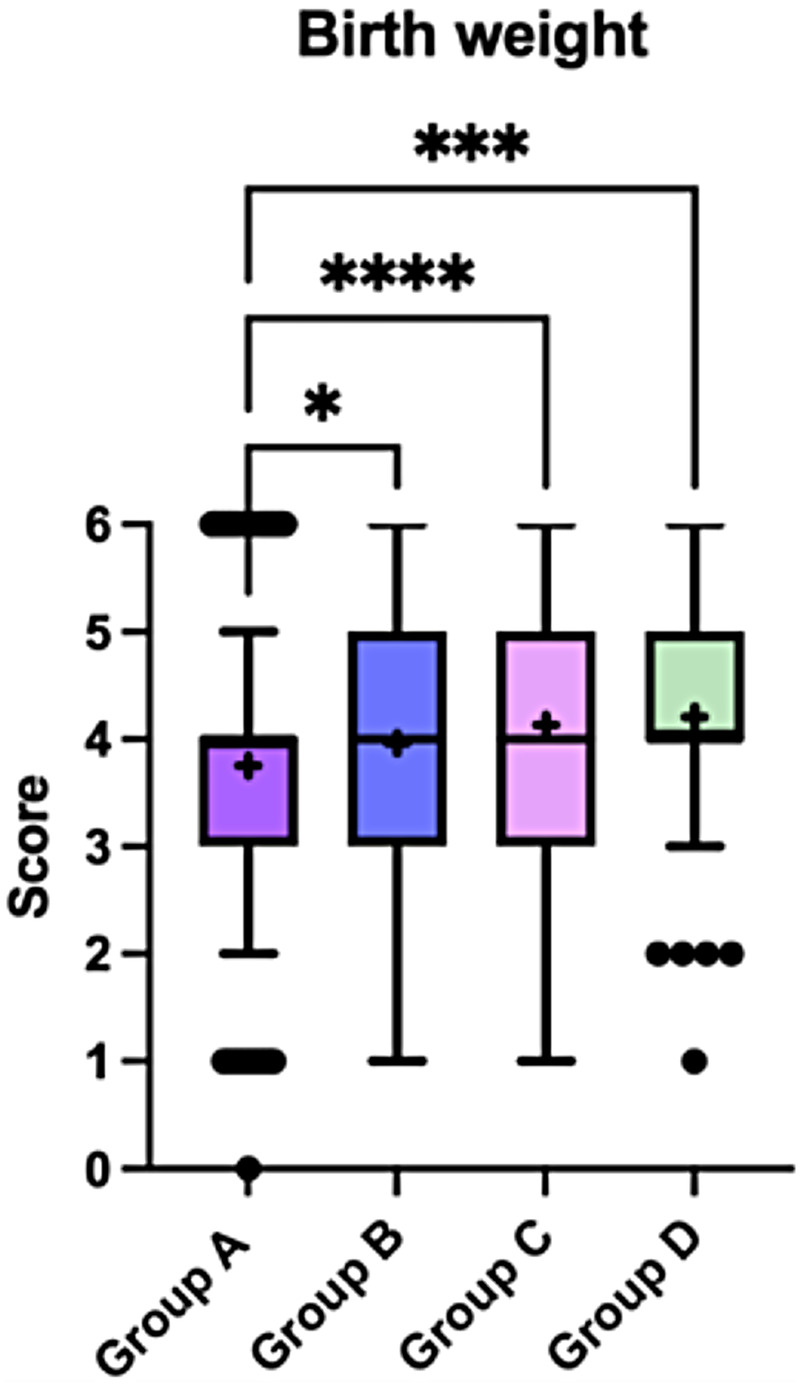
Birth weight across the four child weight-status groups (A–D). Boxplots display the median and interquartile range, with whiskers indicating the minimum and maximum values. Crosses (+) represent mean values, and dots indicate outliers. Statistically significant between-group differences are indicated in the figure (*p* < 0.05). Data were analysed using the Kruskal–Wallis test followed by Dunn's *post-hoc* test with correction for multiple comparisons. Birth weight was coded as an ordinal score: 0 ≤ 1.0 kg; 1 = 1.0–2.0 kg; 2 = 2.0–2.5 kg; 3 = 2.5–3.0 kg; 4 = 3.0–3.5 kg; 5 = 3.5–4.0 kg; 6 ≥ 4.0 kg.

Birth weight differed significantly across weight-status categories. The distribution of birth weight values showed a progressive shift toward higher categories across groups, with a greater proportion of children in Groups B, C, and D falling within the 3.5–4.0 kg birth-weight category, compared with Group A. Specifically, birth weight in this range was reported in 24.0% of children in Group B, 34.3% in Group C, and 32.7% in Group D (Figure 10).

## 4 Discussion

Preschool age represents a sensitive developmental window for the establishment of dietary behaviours, lifestyle patterns, and growth trajectories that may influence long-term health. In this context, the Fair Play a Tavola project provides an updated and comprehensive overview of anthropometric status, eating habits, and lifestyle behaviours among children aged 3–5 years in the Lazio region. By focusing on an age group not currently included in national surveillance systems, this study contributes to the continuity of population-level evidence and reinforces the relevance of preschool age as a critical target for early preventive action in a real-world setting.

Anthropometric screening revealed a prevalence of excess weight affecting 38% of the study population, consistent with previous evidence indicating an early onset of overweight and obesity (23). A progressive increase in waist circumference (WC) was observed across BMI-defined weight-status categories when expressed in absolute centimeters. WC is commonly used in pediatric research as an indicator of abdominal fat distribution, and age and sex specific reference percentiles have been described even in children as young as 2 years (24). However, these reference values primarily describe population distributions and were not developed as validated cardiometabolic risk thresholds for preschool-aged children. In addition, universally accepted cut-offs for waist-to-height ratio (WHtR) or WC-based cardiometabolic risk stratification in children under 6 years are not firmly established. For this reason, neither WHtR nor external WC percentiles were applied as additional risk metrics in this descriptive cross-sectional analysis (25). The observed WC gradient should therefore be interpreted as an anthropometric pattern across BMI categories rather than evidence of established central adiposity or predictive cardiometabolic risk. Longitudinal studies are required to clarify its clinical significance in preschool populations. Although causal inferences cannot be drawn, early growth trajectories, including the timing of adiposity rebound, have been associated with later obesity and metabolic alterations (4). The adiposity rebound, typically occurring between 5 and 6 years of age, represents a critical developmental window; when it occurs earlier, it has been linked to increased obesity risk in later life. From a preventive perspective, identifying early growth deviations may offer an opportunity for timely intervention (26, 27).

Children with overweight or obesity in our sample were more likely to have a higher birth weight, supporting the role of early-life factors in shaping later metabolic outcomes. High birth weight has been associated with increased adiposity and obesity risk across the life course, particularly in the context of intrauterine overnutrition and Large for Gestational Age phenotypes (28, 29). Conversely, low birth weight followed by rapid postnatal catch-up growth has been linked to increased central adiposity, in line with the “thrifty phenotype” hypothesis (30, 31). Together, these findings support a life-course framework in which prenatal and early postnatal factors interact with later environmental exposures.

Parental factors emerged as a relevant contextual element in the interpretation of our findings. A substantial proportion of parents underestimated their child's weight status, even when excess weight was objectively documented, with only partial improvement in awareness in cases of obesity. This perceptual distortion has been consistently reported in the literature and in national surveillance data (32, 33) where 60.7% of mothers of children with overweight and 12.7% of mothers of children with obesity misclassified their child's weight status. These findings confirm that parental misperception is already present before primary school age, representing a major barrier to early prevention. Failure to recognize overweight may delay corrective behaviours and reduce the effectiveness of educational interventions.

In our sample, both maternal and paternal BMI increased progressively across child weight-status categories, in line with previous evidence describing a close association between parental and child weight status (34). This association should be interpreted within a shared familial context, reflecting genetic background, household food environment, and common lifestyle patterns rather than direct causality.

The family constitutes the primary environment in which eating and behavioural habits are formed and consolidated (35, 36). Together with the school environment, which reinforces these lessons through social experiences and educational programs, this family–school synergy represents a key setting for shaping children's dietary quality, self-regulation skills, and long-lasting healthy behaviours (37–42).

Given that parental education influences food quality, nutritional awareness, and children's adoption of healthy lifestyles (37, 43, 44), the higher prevalence of lower educational attainment among parents of children with obesity further underscores the importance of socioeconomic and educational factors from preschool age.

From a behavioural perspective, many children in our sample appeared unlikely to meet age-specific World Health Organization physical activity recommendations. For children aged 3–4 years, WHO 2019 guidelines recommend at least 180 min of total physical activity per day, including at least 60 min of moderate-to-vigorous intensity activity (45). For children aged 5 years and older, the WHO 2020 guidelines recommend an average of at least 60 min per day of moderate-to-vigorous physical activity across the week (46). Given that physical activity in our study was assessed through parent-reported frequency of structured activities rather than total daily movement, these findings should be interpreted cautiously with respect to strict guideline adherence.

Consistent with this pattern, indicators of sedentary behaviour were also common. This is in line with regional OKkio alla Salute data showing that only 55.5% of children limit total screen time to less than 2 h per day. In our younger sample, the majority of children reported no video game use, suggesting that sedentary digital behaviours may still be relatively limited at preschool age.

Regarding sleep, over 80% of the children in our sample fell within the ranges recommended by the World Health Organization for preschool age (10–13 h per day, including naps), likely reflecting the regularity imposed by preschool and family routines. When viewed alongside OKkio alla Salute data, a progressive decline in sleep duration with increasing age becomes apparent, reinforcing the importance of consolidating healthy sleep habits early in life.

With respect to dietary habits, adherence to the CREA Guidelines for Healthy Eating (2018) (22) was only partial, with critical gaps in fruit, vegetable, and cereal intake, which represent the cornerstones of the Mediterranean diet. This limited dietary variety, common at preschool age, may reflect food neophobia and selective eating, which hinder exposure to new foods and slow the acquisition of healthy preferences (7–9, 47–49). In line with OKkio alla Salute findings, low fruit and vegetable consumption appears to be already established during preschool age and to persist into primary school, suggesting early consolidation of these dietary behaviours.

Protein intake, although closer to guidelines, showed qualitative imbalances, with red meat consumed more frequently than recommended. This high-protein pattern has been linked to accelerated growth and fat-mass gain through IGF-1/GH modulation (50). As for sugary drinks, both preschool children and older children showed encouraging adherence to recommendations, with 88% and 62%, respectively, reporting only occasional consumption. Similarly, daily breakfast consumption emerged as a relatively consolidated habit, reported by 77% of preschoolers, in line with regional surveillance data (70.8%), suggesting that this protective behavior may be established early and maintained over time.

Overall, these findings reinforce the relevance of early childhood as a critical phase during which nutritional and lifestyle risk patterns start to take shape, highlighting the value of early observation and prevention.

Beyond its epidemiological findings, Fair Play a Tavola was conceived as a combined educational and screening initiative implemented within routine preschool settings.

Within the present manuscript, the educational component of Fair Play a Tavola should be interpreted as the operational context in which anthropometric screening and behavioural assessment were conducted, rather than as an evaluated intervention. The current analysis reports cross-sectional baseline data collected during program implementation and does not assess behavioural change attributable to the educational session.

From an implementation perspective, the program was implemented across 57 preschools, with delivery of one standardized 60-minute educational session per class and one structured parent feedback meeting per school. Group size was adapted to classroom composition, and sessions were delivered by trained nutritionists following standardized procedures. However, detailed indicators of implementation fidelity, engagement levels, and long-term behavioural impact were not systematically collected and therefore cannot be evaluated within the present analysis. Formal assessment of educational effectiveness would require longitudinal follow-up or controlled study designs.

From a public health perspective, these results support the relevance of future interventions aligned with international recommendations for multidimensional Intensive Health Behavior and Lifestyle Treatment (IHBLT) programs (51), which emphasize the integration of nutritional education, physical activity promotion, and family engagement over sufficient duration (at least 26 h over 3–12 months). Although the present study was not designed to evaluate intervention effectiveness, such integrated models represent the reference framework for future, more structured initiatives aimed at the early prevention of childhood obesity.

Interpretation of these findings should also take into account that questionnaire and anthropometric analyses were restricted to a subsample of children with complete data, which may limit full representativeness of the overall enrolled cohort and the broader preschool population.

### 4.1 Strengths and limitations

This study has several strengths. It includes a large and heterogeneous sample of preschool children recruited from multiple preschools, with anthropometric measurements performed using standardized procedures by trained nutritionists, ensuring internal consistency and data reliability. Importantly, it provides original and updated data on an age group (3–5 years) that is currently not covered by national nutrition surveillance systems in Italy, addressing a critical gap in early childhood prevention. The integration of epidemiological assessment with a structured, school-based nutrition education program, together with the multilingual administration of questionnaires, enhances inclusiveness and real-world applicability. In addition, the close collaboration between schools, families, and healthcare professionals demonstrates that preventive activities can be implemented in preschool settings.

Some limitations inherent to the study design should also be acknowledged. Schools were recruited on a voluntary basis following direct presentation of the project, and no probability-based sampling strategy was adopted; therefore, participating schools constitute a convenience sample. It is possible that schools more interested in health promotion initiatives were more likely to participate, introducing potential self-selection bias at the institutional level. Consequently, findings should be interpreted as descriptive and may not be fully generalizable to all preschool settings in the Lazio region. Furthermore, no area-level socioeconomic indicators were systematically collected at the school level, limiting contextual or stratified analyses according to socioeconomic characteristics.

The cross-sectional nature of the study does not allow causal inference nor evaluation of changes in anthropometric or behavioural outcomes over time. Dietary and lifestyle information was collected through parent-reported questionnaires, which may be affected by recall bias or social desirability bias. Moreover, although the questionnaire was adapted from established surveillance tools and reviewed by experts in paediatric nutrition to ensure face and content validity, it was not formally validated. Data were collected across multiple preschools without adjustment for clustering at the school level, potentially leading to underestimation of variance (52). Analyses were conducted on complete cases only, without imputation of missing data, which may have influenced precision estimates. Finally, the absence of follow-up assessments prevented evaluation of the effectiveness of the educational component on dietary habits and lifestyle behaviours.

## 5 Conclusion

The Fair Play a Tavola project provides a cross-sectional description of anthropometric status and parent-reported dietary and lifestyle behaviours among children aged 3–5 years in the Lazio region, an age group not currently included in national nutritional surveillance systems. A substantial proportion of children presented excess weight according to WHO BMI-for-age standards, together with dietary and behavioural patterns not fully aligned with current recommendations.

Educational activities and parent feedback sessions were implemented alongside screening within preschool settings; however, intervention effectiveness and program acceptability were not formally evaluated, and participation in screening and questionnaire completion was partial. Findings should therefore be interpreted as descriptive of a complete-case subsample.

Overall, these data extend epidemiological knowledge in early childhood and may inform the design of future longitudinal and intervention-based studies aimed at promoting healthy growth trajectories from preschool age within coordinated school and public health strategies.

## Data Availability

The original contributions presented in the study are included in the article/Supplementary Material, further inquiries can be directed to the corresponding author.
