# Supplementary material for: Fair Play a Tavola: a school-based framework integrating nutrition education and anthropometric screening for early identification of excess weight in preschool children
Source: Front Pediatr. 2026 Mar 27;14:1802064. doi: 10.3389/fped.2026.1802064 (PMC13066127; doi:10.3389/fped.2026.1802064)
Supplement: Supplementary file 1 [file Table1.docx]

| **Complete set of anthropometric screening variables and questionnaire items, reported as median (IQR), across the four child weight-status groups.** | | | | | | | | | | | | | |
| --- | --- | --- | --- | --- | --- | --- | --- | --- | --- | --- | --- | --- | --- |
| **Anthropometric characteristics** | | | | | | | | | | | | | |
|  | | | **Group A**  **(median [Q1–Q3])** | | **Group B**  **(median [Q1–Q3])** | | **Group C**  **(median [Q1–Q3])** | | **Group D**  **(median [Q1–Q3])** | | **p–value (K–W)** | | **Effect size (ε² magnitude)** |
| Waist circumference | | | 51 (49.0–53.0) | | 53 (51.0–55.0) | | 56 (53.4–58.0) | | 61 (58.0–65.5) | | < 0.0001 | | 0.33 (large) |
| **Structured physical activity** | | | | | | | | | | | | | |
|  | | **Group A**  **(median [Q1–Q3])** | | | **Group B**  **(median [Q1–Q3])** | | **Group C**  **(median [Q1–Q3])** | | **Group D**  **(median [Q1–Q3])** | | **p–value (K–W)** | | **Effect size (ε² magnitude)** |
| Physical activity | | 1 (1–2) | | | 1 (0–2) | | 1 (0–2) | | 2 (0–2) | | 0.0213 | | 0.00 (negligible) |
| **Use of technology devices** | | | | | | | | | | | | | |
|  | | **Group A**  **(median [Q1–Q3])** | | | **Group B**  **(median [Q1–Q3])** | | **Group C**  **(median [Q1–Q3])** | | **Group D**  **(median [Q1–Q3])** | | **p–value (K–W)** | | **Effect size (ε² magnitude)** |
| Videogames – school days | | 0 (0–0) | | | 0 (0–0) | | 0 (0–0) | | 0 (0–1) | | 0.0330 | | 0.00 (negligible) |
| Videogames –weekend | | 0 (0–1) | | | 0 (0–1) | | 0 (0–1) | | 0 (0–1) | | 0.0174 | | 0.00 (negligible) |
| TV – school days | | 1 (1–1) | | | 1 (1–1) | | 1 (1–1) | | 1 (1–1) | | ns | | 0.00 (negligible) |
| TV – weekend | | 1 (1–1) | | | 1 (1–1) | | 1 (1–1) | | 1 (1–1) | | ns | | 0.00 (negligible) |
| **Sleep habits** | | | | | | | | | | | | | |
|  | | **Group A**  **(median [Q1–Q3])** | | | **Group B**  **(median [Q1–Q3])** | | **Group C**  **(median [Q1–Q3])** | | **Group D**  **(median [Q1–Q3])** | | **p–value (K–W)** | | **Effect size (ε² magnitude)** |
| Bedtime | | 3 (2–3) | | | 3 (2–3) | | 3 (2–3) | | 3 (2–3) | | ns | | 0.00 (negligible) |
| Wake–up time | | 3 (2–3) | | | 3 (3–3) | | 3 (3–3) | | 3(3–3) | | ns | | 0.00 (negligible) |
| **Eating habits** | | | | | | | | | | | | | |
|  | **Group A**  **(median [Q1–Q3])** | | | **Group B**  **(median [Q1–Q3])** | | **Group C**  **(median [Q1–Q3])** | | **Group D**  **(median [Q1–Q3])** | | **p–value (K–W)** | | **Effect size (ε² magnitude)** | |
| Daily water intake | 3 (1–3) | | | 2 (1–3) | | 2 (1–3) | | 2 (1–3) | | ns | | 0.00 (negligible) | |
| Breakfast consumption | 1 (1–1) | | | 1 (1–1) | | 1 (1–1) | | 1 (1–1) | | ns | | 0.00 (negligible) | |
| Pizza | 1 (1–2) | | | 1 (1–2) | | 1 (1–2) | | 1 (1–2) | | ns | | 0.00 (negligible) | |
| Bread | 3 (2–4) | | | 3 (2–3) | | 2 (2–3) | | 3 (2–3) | | ns | | 0.00 (negligible) | |
| Pasta or other cereals | 3 (2–4) | | | 3 (2–4) | | 3 (2–4) | | 3 (2–4) | | 0.0359 | | 0.00 (negligible) | |
| Red meat | 2 (1–2) | | | 2 (1–2) | | 2 (1.75–2) | | 2 (2–2) | | ns | | 0.00 (negligible) | |
| White meat | 2 (2–3) | | | 2 (2–3) | | 2 (2–2) | | 2 (2–3) | | ns | | 0.00 (negligible) | |
| Fish | 2 (2–2) | | | 2 (2–2) | | 2 (1–2) | | 2 (1.5–2) | | 0.0292 | | 0.00 (negligible) | |
| Eggs | 2 (2–2) | | | 2 (2–2) | | 2 (1–2) | | 2 (1–2) | | 0.0393 | | 0.00 (negligible) | |
| Cold cuts | 1 (1–2) | | | 2 (1–2) | | 1 (1–2) | | 1 (1–2) | | 0.0234 | | 0.00 (negligible) | |
| Fresh cheeses | 2 (1–2) | | | 2 (1–2) | | 2 (1–2) | | 2 (1.5–2) | | ns | | 0.00 (negligible) | |
| Aged cheeses | 2 (2–3) | | | 2 (2–3) | | 2 (2–3) | | 2 (1–2.5) | | ns | | 0.00 (negligible) | |
| Legumes | 2 (1–2) | | | 2 (1–2) | | 2 (1–2) | | 2 (1–2) | | ns | | 0.00 (negligible) | |
| Yogurt | 2 (1–3) | | | 2 (1–3) | | 2 (1–2) | | 2 (1–3) | | ns | | 0.00 (negligible) | |
| Milk | 4 (2–4) | | | 4 (2–4) | | 4 (2–4) | | 4 (2–4) | | ns | | 0.00 (negligible) | |
| Vegetable drinks | 0 (0–0) | | | 0 (0–0) | | 0 (0–0) | | 0 (0–0) | | 0.0290 | | 0.00 (negligible) | |
| Nuts | 1 (0–1) | | | 1 (0–1) | | 0 (0–1) | | 0 (0–1) | | ns | | 0.00 (negligible) | |
| Vegetables | 2 (2–4) | | | 3 (2–4) | | 3 (2–3) | | 2 (2–4) | | ns | | 0.00 (negligible) | |
| Fruits | 3 (2–4) | | | 3 (2–4) | | 3 (2–4) | | 3 (2–4.5) | | ns | | 0.00 (negligible) | |
| E.V.O. Oil | 4 (3–5) | | | 4 (3–5) | | 4 (3–5) | | 4 (3–5) | | 0.0325 | | 0.00 (negligible) | |
| Seed Oil | 0 (0–1) | | | 0 (0–1) | | 0 (0–1) | | 0 (0–1) | | ns | | 0.00 (negligible) | |
| Butter | 0 (0–1) | | | 0 (0–1) | | 0 (0–1) | | 0 (0–1) | | ns | | 0.00 (negligible) | |
| Snacks | 2 (2–3) | | | 2 (2–3) | | 2 (2–3) | | 2 (2–3) | | ns | | 0.00 (negligible) | |
| Fast food or ethnic food | 0 (0–1) | | | 0 (0–1) | | 0 (0–1) | | 1 (0–1) | | 0.0218 | | 0.00 (negligible) | |
| Chocolate | 2 (1–2) | | | 2 (1–2) | | 2 (1–2) | | 2 (1–2) | | ns | | 0.00 (negligible) | |
| Fruit juice | 2 (1–2) | | | 2 (1–2) | | 2 (1–2) | | 2 (0.5–2) | | ns | | 0.00 (negligible) | |
| Sweet sugar beverages | 0 (0–1) | | | 0 (0–1) | | 0 (0–1) | | 0 (0–1) | | 0.0467 | | 0.00 (negligible) | |
| **Family assessment** | | | | | | | | | | | | | |
|  | **Group A**  **(median [Q1–Q3])** | | | **Group B**  **(median [Q1–Q3])** | | **Group C**  **(median [Q1–Q3])** | | **Group D**  **(median [Q1–Q3])** | | **p–value (K–W)** | | **Effect size (ε² magnitude)** | |
| Parental perception BMI | 1 (1–1) | | | 1 (1–1) | | 1 (1–1) | | 1 (1–2) | | < 0.0001 | | 0.16 (large) | |
| First parent’s educational attainment | 4 (3 –4) | | | 4 (3 –4) | | 4 (3 –4) | | 3 (3 –4) | | ns | | 0.00 (negligible) | |
| Second parent’s educational attainment | 3 (3–4) | | | 4 (3–4) | | 3 (3–4) | | 3 (3–4) | | 0.0460 | | 0.00 (negligible) | |
| Employment type | 1 (1–2) | | | 1 (1–2) | | 1 (1–2) | | 1 (1–2) | | ns | | 0.00 (negligible) | |
| Maternal BMI | 22 (20.0–23.9) | | | 22.2 (20.2–25.0) | | 23 (21.3–24.9) | | 24.5 (22.3–27.5) | | < 0.0001 | | 0.04 (small) | |
| Paternal BMI | 25 (23.0–27.0) | | | 25.2 (24.0 –27.0) | | 25.9 (24.0–28.0) | | 26.3 (24.7–29.0) | | < 0.0001 | | 0.02 (small) | |
| Family medical condition | 1 (0–3) | | | 1 (0–3) | | 1 (0–3) | | 2 (0–3) | | ns | | 0.00 (negligible) | |
| Child’s medical condition | 0 (0–0) | | | 0 (0–0) | | 0 (0–0) | | 0 (0–0) | | ns | | 0.00 (negligible) | |
| Child’s food allergies and intolerances | 0 (0–0) | | | 0 (0–0) | | 0 (0–0) | | 0 (0–0) | | ns | | 0.00 (negligible) | |
| **Neonatal information** | | | | | | | | | | | | | |
|  | **Group A**  **(median [Q1–Q3])** | | | **Group B**  **(median [Q1–Q3])** | | **Group C**  **(median [Q1–Q3])** | | **Group D**  **(median [Q1–Q3])** | | **p–value (K–W)** | | **Effect size (ε² magnitude)** | |
| Type of delivery | 0 (0–1) | | | 0 (0–1) | | 0 (0–1) | | 0 (0–1) | | ns | | 0.00 (negligible) | |
| Birth details | 2 (2–2) | | | 2 (2–2) | | 2 (2–2) | | 2 (2–2) | | ns | | 0.00 (negligible) | |
| Birth weight | 4 (3–4) | | | 4 (3–5) | | 4 (3–5) | | 4 (4–5) | | < 0.0001 | | 0.02 (small) | |
| Type of feeding | 8 (4–8) | | | 8 (4–8) | | 8 (4–8) | | 7 (2–8) | | ns | | 0.00 (negligible) | |

**Table 1** Screening- and questionnaire-derived variables across the four child weight-status groups (A–D), grouped by thematic domain. Data are reported as median and interquartile range (IQR), unless otherwise specified. Between-group comparisons were performed using the Kruskal–Wallis test, followed by Dunn’s post-hoc test with correction for multiple comparisons. Effect sizes for the Kruskal–Wallis test are reported as epsilon-squared (ε²) and interpreted according to conventional thresholds (negligible, small, moderate, large). Statistically significant differences are indicated where applicable (p < 0.05).

| **Family characteristics of the study sample derived from the screening questionnaire.** | | | | | | | | | | | |
| --- | --- | --- | --- | --- | --- | --- | --- | --- | --- | --- | --- |
| **Parents’ educational attainment – Responding parent, n (%)*** | | | | | | | | | | | |
| **No formal education** | | | **Primary education** | | **Lower secondary education** | | | **Upper secondary education** | **University degree** | | **Postgraduate degree** |
| 1.2% | | | 0.1% | | 7.3% | | | 33.5% | 36.3% | | 21.6% |
| **Parents’ educational attainment – Other parent, n (%)†** | | | | | | | | | | | |
| **No formal education** | | **Primary education** | | | | **Lower secondary education** | | **Upper secondary education** | **University degree** | | **Postgraduate degree** |
| 0.6% | 0.1% | | | | | | 12.2% | 40.2% | 30.9% | | 16.0% |
| **Employment outside the home (responding parent), n (%)** | | | | | | | | | | | |
| **No** | | | | | **Part-time** | | | | **Full-time** | | |
| 21.3% | | | | | 28.6% | | | | 50.1% | | |
| **Parental weight status** | | | | | | | | | | | |
| **Maternal BMI,**  **mean** $\boldsymbol{\pm}$**SD** | | | | **Maternal Overweight/obesity**  **(BMI** $\boldsymbol{\geq}$**25 kg/m^2^), n (%)** | | | | **Paternal BMI,**  **mean** $\boldsymbol{\pm}$**SD** | | **Paternal Overweight/obesity**  **(BMI** $\boldsymbol{\geq}$**25 kg/m^2^), n (%)** | |
| 22.84 $\pm$ 3.87 | | | | 21.6% | | | | 26.00 $\pm$3.00 | | 49.9% | |
| **Family history of chronic diseases, n (%)** | | | | | | | | | | | |
| **Type 1 diabetes** | | | **Type 2 diabetes** | | **Cardiovascular diseases** | | | **Obesity** | **Thyroid disorders** | | **No reported conditions** |
| 3.2% | | | 21.0% | | 29.9% | | | 5.6% | 7.5% | | 32.8% |

*Responding parent: n = 1542

† Percentages for the other parent are calculated excluding single-parent households (n = 1528).

**Table 2** Family characteristics of the study sample derived from the screening questionnaire, grouped by thematic domain. Variables include parental educational attainment, employment outside the home, parental weight status, and family history of chronic diseases. Data are presented as n (%) or mean ± standard deviation (SD), as appropriate. Overweight/obesity was defined as BMI ≥ 25 kg/m².
